# Progress in Fevers

**Published:** 1901-06-08

**Authors:** 


### PROGRESS IN FEVERS.

(*Continued from page* 148.)

**Prophylaxis in malaria** Manson arranges under three heads:—Destruction of mosquitos, prevention of infection of mosquitos and prevention of mosquito bites. For the suppression of mosquitos improved drainage, reclamation of swamps, and avoidance of unnecessary puddles where *Anopheles* can breed are required. "Painting" the water with petroleum frees it from mosquito larvæ, but the operation has to be frequently renewed. Prevention of mosquito infection might be attained by all-round drugging with quinine as Koch suggests, but this is perfectly impracticable. In this connection H. E. Durham[40] points out that although quinine kills the asexual parasite it does not kill the sexual forms, at any rate rapidly. Protection of malarial patients from being bitten should be scrupulously observed, and deportation to a locality free from *Anopheles* has been advocated. W. N. Berkeley[41] considers malaria should be notified, and inspection of infected houses made compulsory in order to ensure the destruction of mosquitos and the isolation of patients. Several observers, especially in tropical Africa, point to the native child as the great source of malarial infection. Christophers and Stephens[42] find that *Anopheles* congregates whenever a clearing with native dwellings occurs. For Europeans settlement a mile from a native village would suffice. "To stamp out native malaria is at present chimerical." Malaria in the native child may present none of the characteristic signs of an attack of fever. Daniels[43] emphasises with equal force the danger of Europeans and natives living close together in a malarial country. He has also made postmortem investigations[44] into the presence of malarial pigment in children and adults, and concludes that children acquire immunity probably by a previous attack.

Investigations made by the members of the Liverpool Malaria Expedition to Nigeria[45] show how prevalent is the parasite in native children, and how a European factory may be practically fever free if far enough removed from a native settlement, although situated in a brackish mangrove swamp swarming with *Anopheles*. The freedom from malaria afforded by protection from mosquito bites has been shown in the Campagna experiments. Sir William MacGregor[46] points the moral of all the new teaching on malaria, and shows how administrators, school teachers, hospital authorities, nurses, and the general public must all reckon with this disease, and by administration, teaching, and organisation seek to minimise the chances of infection and exposure. Leichman[47] recommends a new method of applying Romanowsky's stain for the detection of the malarial parasite, which is approximately that of Maurer. The advantages claimed are that in all cases of tertian fever the infected red corpuscles show ruby red dots (Schüffner's dots), very young parasites and mixed infections are readily detected, and the method is simple. Thin cover-glass films of blood are fixed in absolute alcohol and ether, washed, and dried. Weak watery solutions of eosin and of alkaline methylene blue are then poured simultaneously on the film. The nuclei of the leucocytes, the blood platelets, and in cases of tertian fever Schüffner's dots, are stained bright ruby red.

**Blackwater fever.**—Recurrent rigors, fever, vomiting, icterus, and hæmoglobinuria are the main symptoms of blackwater fever as pointed out by Sambon.[48] Second attacks are common. Rapid destruction of red corpuscles takes place in the blood. The disease is endemic in low swampy grounds, but has a wide distribution elsewhere. All ages and both sexes may suffer, but men of middle age are oftenest attacked. As regards etiology three theories prevail:—1. *The "malarial" theory*: this Sambon discards. Occasionally a few malarial parasites are found, but these are accidentally present and disappear during the fever. 2. *The quinine theory* he also discards. The connection of quinine with blackwater fever is merely one of coincidence. 3. *The specific theory*: Manson advocated the specific origin of blackwater fever in 1893, and Sambon thinks it may be due to a protozoal organism. Treatment must be symptomatic: water should be freely given; quinine generally does harm.

[40] Brit. Med. Jour., March 2. [41] Med. Rec., Jan. 26. [42] Brit. Med. Jour., Jan. 26, p. 240. [43] Ibid. [44] Lancet, Dec. 15, p. 1732. [45] Brit. Med. Jour., Jan. 12. [46] Lancet, Oct. 16. [47] Brit. Med. Jour., Mar. 16. [48] Practitioner, March.

